# Ancestral remnants or peripheral segregates? Phylogenetic relationships of two narrowly endemic *Euphrasia* species (Orobanchaceae) from the eastern European Alps

**DOI:** 10.1093/aobpla/plz007

**Published:** 2019-02-19

**Authors:** Da Pan, Peter Schönswetter, Tim Moser, Ernst Vitek, Gerald M Schneeweiss

**Affiliations:** 1 Department of Botany and Biodiversity Research, University of Vienna, Vienna, Austria; 2 Institute of Botany, University of Innsbruck, Innsbruck, Austria; 3 Department of Botany, Natural History Museum, Vienna, Austria

**Keywords:** Alps, endemics, *Euphrasia*, peripheral segregate, polyploidy

## Abstract

Endemism in mountain ranges is considered to be the result of a number of factors, including restriction to refugia during Pleistocene climate fluctuations. However, isolation in glacial refugia cannot explain the origin of narrowly endemic taxa restricted to formerly heavily glaciated areas. Here, we investigate the phylogeny of two narrowly endemic species, *Euphrasia inopinata* and *E. sinuata* (Orobanchaceae), found exclusively in formerly heavily glaciated areas of the eastern European Alps. As both species are diploid and very similar to the widespread (allo)polyploid *E. minima*, we test whether the restricted distributions of *E. inopinata* and *E. sinuata* are relictual, i.e. the two species are ancestral diploid remnants of a polyploid complex, or whether they are derived, i.e. the two species are peripheral segregates of a more widespread diploid. Based on internal transcribed spacer (ITS) sequence and amplified fragment length polymorphism (AFLP) fingerprint data it is shown that *E. inopinata* and *E. sinuata*, whose diploid ploidy level is confirmed for all analysed individuals via flow cytometry, are phylogenetically closely related to diploid *E. alpina* s. l. (series *Alpinae*) instead of *E. minima* (series *Parviflorae*). In addition, there is no evidence that these two diploid species participated in the formation of allotetraploid *E. minima*. Thus, *E. inopinata* and *E. sinuata* are interpreted as peripheral segregates of the widespread *E. alpina* s. l. Shifts in pollination system from allogamy in *E. alpina* s. l. to autogamy in *E. inopinata* and *E. sinuata*, genetic drift in small populations and geographic isolation at the periphery of the range of *E. alpina* s. str. probably contributed to the morphological and ecological differentiation of *E. inopinata* and *E. sinuata*.

## Teaser

The origin of range-restricted diploid species confined to formerly heavily glaciated areas is little understood. A suitable system to address this question is a group of two autogamous eyebright species, *Euphrasia inopinata* and *E. sinuata*, each known only from a few sites in the eastern European Alps. Previous hypotheses suggested these species to be either remnants of the diploid ancestor of the equally autogamous but widespread and polyploid *E. minima* or as peripheral segregates of a more widespread allogamous diploid species.

## Introduction

Mountain ranges host a considerable number of endemic species (Barthlott *et al.* 1996; Körner 2003), which is considered to be the result of the complex origin and histories of mountain ranges, being shaped by geographic isolation, climate changes and strong microhabitat differentiation (Körner 1995; Agakhanjanz and Breckle 1995). Distribution areas of narrowly endemic species, for instance in the European Alps (hereinafter simply referred to as Alps), often coincide with Pleistocene refugia (Tribsch and Schönswetter 2003; Tribsch 2004), suggesting that Pleistocene climate fluctuations had major impacts on the biogeography of endemic taxa. For narrowly endemic taxa restricted to formerly heavily glaciated areas, however, alternative explanations have to be sought. These include *in situ* survival on nunataks, survival in periglacial areas and extirpation in those source areas due to postglacial environmental changes, or rapid *in situ* speciation of postglacial (re)colonizers (Kolář *et al.* 2013).

**Figure 1.**
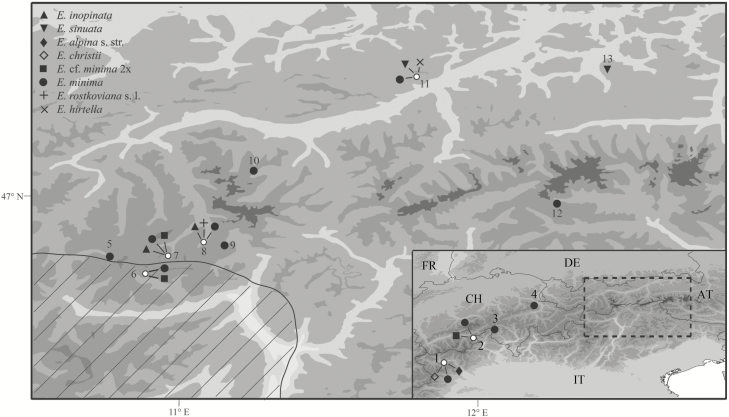
Locations of sampled populations of the investigated *Euphrasia* species (see Table 1 and **Supporting Information—Table S1** for detailed information). The hatched area indicates the north-eastern edge of the distribution range of *Euphrasia alpina* s. str. (Vitek 1985, modified using data from http://florafauna.it/). The insert shows sampled locations in the western Alps as well as the position of the sampling area shown in the main map. Country abbreviations: AT, Austria; CH, Switzerland; DE, Germany; FR, France; IT, Italy.

Members of the genus *Euphrasia* (Orobanchaceae) from the eastern Alps form a group well suited to study hypotheses on the origin of narrowly distributed species in formerly strongly glaciated areas. Apart from several widely distributed species, *Euphrasia* in this region includes two locally endemic species, *E. inopinata* restricted to a few populations in the central Alps (Ötztaler Alpen, Tyrol) and *E. sinuata* found in two disjoint areas in the northern calcareous Alps (Rofan Mountains, Tyrol) and in the central Alps (Kitzbühler Alpen, Tyrol; Fig. 1; Ehrendorfer and Vitek 1984). Both species are found in the subalpine and lower alpine zone, but differ edaphically as *E. inopinata* occurs on siliceous substrate, whereas *E. sinuata* grows on limestone and dolomites. As the current distribution areas of these species are not associated with any peripheral glacial refugium (Schönswetter *et al.* 2005), Ehrendorfer and Vitek (1984) suggested nunatak survival during the last glacial period for both species.

**Table 1.** Sampling locations of investigated *Euphrasia* species (**see****Supporting Information—Table S1** for detailed information).

Species	Location no.	Region^1^	Latitude/longitude	Herbarium
*E. minima*	1	I, Alpi Graie: Vallone di Laures	45°41′13″/7°24′25″	WU:GMS-266
	2	CH, Alpi Lepontine: Gruppo del Monte Leone	46°15′27″/8°03′57″	WU:GMS-267
	3	CH, Alpi Lepontine: Alpi Ticinesi	46°26′41″/8°30′15″	WU:GMS-269
	4	CH, Glarner Alpen	46°58′18″/9°23′50″	WU:GMS-268
	5	I, Alpi Venoste/Ötztaler Alpen	46°49′12″/10°41′53″	WU:GMS-270
	6	I, Alpi Venoste/Ötztaler Alpen	46°45′20″/10°49′09″	WU:GMS-264
	7	A, Ötztaler Alpen	46°49′07″/10°54′02″	WU:GMS-256
	8	A, Ötztaler Alpen	47°51′57″/11°01′24″	W:NHM2014-0014158
	9	I, Alpi Venoste/Ötztaler Alpen	46°51′23″/11°05′37″	WU:GMS-261
	10	A, Stubaier Alpen	47°06′57″/11°11′49″	WU:GMS-273
	11	A, Rofangebirge und Brandenberger Alpen	47°26′37″/11°45′54″	WU:GMS-274
	12	A, Hohe Tauern: Venedigergruppe and Lasörlinggruppe	47°00′04″/12°15′10″	WU:GMS-272
*E. sinuata*	11	A, Rofangebirge und Brandenberger Alpen	47°26′37″/11°45′54″	WU:GMS-251
	13	A, Kitzbüheler Alpen	47°28′22″/12°25′49″	W:NHM2014-0014161
*E. inopinata*	7	A, Ötztaler Alpen	46°49′07″/10°54′02″	WU:GMS-249
	8	A, Ötztaler Alpen	47°51′57″/11°01′24″	W:NHM2014-0014157
*E.* cf. *minima* 2x	2	CH, Alpi Lepontine: Gruppo del Monte Leone	46°15′27″/8°03′57″	WU:GMS-255
	6	I, Alpi Venoste/Ötztaler Alpen	46°45′20″/10°49′09″	WU:GMS-254
	7	A, Ötztaler Alpen	46°49′07″/10°54′02″	WU:GMS-253
*E. alpina* s. str.	1	I, Alpi Graie: Vallone di Laures	45°41′13″/7°24′25″	WU:GMS-276
*E. christii*	1	I, Alpi Graie: Vallone di Laures	45°41′13″/7°24′25″	WU:GMS-277
*E. rostkoviana* s. l.	8	A, Ötztaler Alpen	46°51′57″/11°01′24″	W:NHM2014-0014158
*E. hirtella*	11	A, Rofangebirge und Brandenberger Alpen	47°26′37″/11°45′54″	W:NHM2014-0014155

^1^I = Italy; CH = Switzerland; A = Austria.

Both *E. inopinata* and *E. sinuata* are taxonomically assigned to series *Parviflorae* (Ehrendorfer and Vitek 1984), which otherwise in the study region only includes tetraploids (Yeo 1978). Thus, the two diploid species might actually be relics of formerly more widely distributed diploids that have been largely replaced by tetraploids, i.e. in the Alps by the morphologically and ecologically extremely plastic *E. minima* (Vitek 1998). Based on morphological data, it has been suggested that the diploids *E. inopinata* and *E. sinuata* were involved in the formation of *E. minima*, a presumable allopolyploid (Yeo 1978), either directly as one of the parents (Ehrendorfer and Vitek 1984) or indirectly via introgression into an allotetraploid derivative of *E. alpina* subsp. *christii* (from series *Alpinae*) and *E. hirtella* (from series *Euphrasia*, formerly, nomenclaturally incorrectly, named series *Grandiflorae*), transferring the character of small flowers to *E. minima* (Vitek 1986). An alternative hypothesis is that *E. inopinata* and *E. sinuata* are the result of the evolution of high-elevation dwarf forms from other large-flowered diploids, such as *E. alpina* subsp. *alpina* (series *Alpinae*; Fig. 7 in Vitek 1986). As morphological traits in *Euphrasia* are, however, variable and ecologically convergent (Yeo 1978; Vitek 1998; Twyford *et al.* 2018), hypotheses derived on the basis of phenotypical comparison can be potentially misleading. Hence, for elucidating the taxonomic position and evolution of *E. inopinata* and *E. sinuata* molecular data are needed.

Here we study phylogenetic relationships of the two narrow endemics *E. inopinata* and *E. sinuata* in the eastern Alps. To this end, we use nuclear DNA sequences from the internal transcribed spacer (ITS) of the rRNA operon as well as the amplified fragment length polymorphism (AFLP) fingerprinting method (Vos *et al.* 1995). Nuclear ITS has been recently used for DNA barcoding of British *Euphrasia* taxa (Wang *et al.* 2018), and although not resolving at the species level ITS sequences allow series *Euphrasia* and *Alpinae* to be unambiguously distinguished. Amplified fragment length polymorphisms have several advantages, including whole genome coverage, bi-parental inheritance and independence from prior sequence information, and have also been widely used for polyploid plants (e.g. Hedrén *et al.* 2001; Guo *et al.* 2005; Dixon *et al.* 2009; Pachschwöll *et al.* 2015; Winkler *et al.* 2017). Specifically, we want to test the hypotheses (i) that *E. inopinata* and/or *E. sinuata* are parental taxa of the allotetraploid *E. minima* (i.e. an origin of *E. minima* from *E. inopinata*/*E. sinuata ×* species from series *Euphrasia*, specifically *E. officinalis* or *E. hirtella*) and (ii) that they are segregates from more widespread diploid species not involved in the origin of *E. minima*, which instead might have originated from a crossing between *E. alpina* subsp. *christii* and *E. hirtella*.

## Methods

### Studied species and sampling

None of the investigated taxa is protected and none of the sampled populations is located in protected areas. Whole individuals from *E. inopinata*, *E. sinuata*, *E. alpina* subsp. *alpina*, *E. alpina* subsp. *christii* (the latter two jointly referred to as *E. alpina* s. l.), *E. officinalis* subsp. *rostkoviana*, *E. officinalis* subsp. *picta* (the latter two jointly referred to as *E. officinalis* s. l.) and *E. hirtella* were collected and stored in silica gel during 2015 and 2016 (Fig. 1; Table 1; **see****Supporting Information—Table S1**). Per sampling site, whose size did not exceed 1 m^2^, 1–18 individuals were collected. As *E. inopinata* and *E. sinuata* are consistently very small in all parts (Ehrendorfer and Vitek 1984) and may be confused with small-flowered forms of *E. minima*, we explicitly targeted individuals with relatively small flower size to increase the chance to recover diploids. Herbarium vouchers are deposited in the Natural History Museum Vienna (W) and the University of Vienna (WU); however, due to the dwarfish size of many of the sampled individuals, after genome size measurement and AFLP fingerprinting no material was left to be used as herbarium voucher; for those photographic images are provided as vouchers **[see****Supporting Information—Table S1****]**.

### Ploidy level determination

Flow cytometry (FCM) of 4′,6-diamidino-2-phenylindole (DAPI)-stained nuclei was used for estimation of relative DNA content (Suda and Trávníček 2006) from silica gel-dried samples. Similar amounts of desiccated sample and fresh internal reference standard were combined in a Petri dish containing 0.5 mL of cold (5–10 °C) Otto I buffer (0.1 M citric acid, 0.5 % Tween 20) and chopped with a razor blade. *Bellis perennis* (2C = 3.38 pg; Schönswetter *et al.* 2007) was selected as primary reference standard (Doležel *et al.* 1998). After filtration through a 42 µm nylon mesh, samples were stained for 10 min at room temperature in a solution containing 1 mL of Otto II buffer (0.4 M Na_2_HPO_4_·12H_2_O), 2-mercaptoethanol and DAPI at final concentrations of 4 µg mL^−1^. The relative fluorescence intensity of 3000 particles was recorded using a Partec PA II flow cytometer (Partec, Münster, Germany) equipped with an HBO mercury arc lamp after incubation for 5 min at room temperature. If the coefficient of variation (CV) of the G0/G1 peak of a sample exceeded the 5 % threshold, the analysis was discarded and the sample re-measured.

### DNA sequencing and AFLP fingerprinting

Total genomic DNA was extracted from similar amounts of silica-dried tissue (ca. 5 mg) applying a CTAB protocol (Doyle and Doyle 1987) with modifications (Jang *et al.* 2013). Internal transcribed spacer sequences were amplified using the primers ITS4 and ITS5 from White *et al.* (1990). The PCR reaction mix (10 µL in total) contained 5 µL ReddyMix PCR Master Mix (Thermo Fisher Scientific, Vienna, Austria), 1.6 µL Trehalose Dihydrate (1 M; Sigma, Vienna, Austria), 0.1 µL bovine serum albumin (BSA) (20 mg mL^−1^, Thermo Fisher Scientific), 0.3 µL dimethyl sulfoxide (DMSO) (100 %, Thermo Fisher Scientific), 1 µL of each primer (5 µM µL^−1^) and 1 µL of genomic DNA with unknown concentration. The PCR condition was: denaturation for 2 min at 94 °C; 35 cycles each of 30 s at 95 °C, 30 s at 94 °C, 1 min at 72 °C; 2 min at 72 °C. PCR products were checked on a 1.5 % agarose gel. PCR products were purified using the mixture EXO/FastAP (Exonuclease I and Thermosensitive Alkaline Phosphatase, Thermo Fisher Scientific) following the manufacturer’s instructions. For each sequencing reaction, a mixture was made with 5 µL of purified PCR product, 0.4 µL of BigDye terminator V3.1 (Applied Biosystems, Foster City, CA, USA), 1.8 µL of BigDye buffer (5×, Applied Biosystems), 1 µL of primer (5 µM µL^−1^), 2 µL of Trehalose Dihydrate (1 M, Sigma) and 2.8 µL of water (Alfa Aesar, Karlsruhe, Germany). Cycle-sequencing products were sequenced on an ABI 3730 DNA Analyzer capillary sequencer (Applied Biosystems).

Amplified fragment length polymorphism fingerprinting was performed with the following selective primer combinations (fluorescent dye in brackets): EcoRI (6-FAM)-ATG/MseICTT, EcoRI (VIC)-ACG/MseI-CAA and EcoRI (NED)-AGC/MseI-CTG. In two samples, DNA was replaced by water to test for systematic contamination. In addition, 10 samples were replicated in order to calculate the error rate using AFLPTools (https://github.com/geneva/AFLPTools). The AFLP laboratory procedure followed that of Rešetnik *et al.* (2014).

### Data analyses

Internal transcribed spacer sequences were assembled and edited using SeqMan II 5.05 (DNASTAR Inc., Madison, WI, USA). Sequences downloaded from GenBank (Gussarova *et al.* 2008; Wang *et al.* 2018) were combined with our newly obtained sequences and aligned using MUSCLE (https://www.ebi.ac.uk/Tools/msa/muscle/; Edgar 2004). The best-fit substitution model was selected using jModelTest 2.1.10 (Darriba *et al.* 2012) based on the Akaike information criterion (AIC). The general time reversible (GTR) model with gamma-distributed substitution rates was selected for our data set. Maximum likelihood (ML) analysis was done using RAxML 8.2.3 (Stamatakis 2014) employing the fast bootstrap approach (Stamatakis et al. 2008) with 1000 replicates. Bayesian inference (BI) was conducted using MrBayes 3.2.7 (Ronquist *et al.* 2012), employing four independent Markov chain Monte Carlo (MCMC) of 2 × 10^7^ generations and sampling trees every 5000 generations. Chain convergence was assessed by visual inspection of the traces and via effective sample size (ESS) values calculated with Tracer 1.6 (Rambaut *et al.* 2018). As in all cases ESS values were safely above 200, the four runs were combined after discarding the first 10 % of each MCMC as burn-in.

Raw AFLP electropherograms were analysed in PeakScanner 1.0 (ABI, Foster City, CA, USA) with default parameters. Automatic scoring was performed in R (R Core Team 2011) using the package RawGeno 2.0 (Arrigo *et al.* 2009). Based on visual inspection of the electropherograms with Genographer 1.6 (formerly available at http://hordeum.oscs.montana.edu/genographer), the scored range was set to 140–500 bp. Nineteen individuals were excluded because at least one of the primer combinations did not work well. Minimum reproducibility of each marker was set to 85 %. Minimum bin width and maximum bin width were left at the default of 1 and 1.5 bp, respectively.

To infer the genetic relationships among individuals from AFLP data, a neighbour-net analysis (Bryant and Moulton 2004) using Jaccard distances was performed in SplitsTree 4.14.2 (Huson and Bryant 2006). Additionally, the genetic structure among samples was analysed via a principal coordinate analysis (PCoA) on the basis of Jaccard distances using the R package vegan (Oksanen *et al.* 2013) and via the Bayesian clustering approach implemented in Structure 2.3.4 (Pritchard *et al.* 2000). For the Structure analysis, the admixture model with correlated allele frequencies was used. The maximal number of groups (*K*) was set to 1–10. The run length of the MCMC was 10^6^ iterations with additional 10^5^ iterations as burn-in; each run was replicated 10 times. The best *K* was chosen based on Delta *K* (Evanno *et al.* 2005) using Structure Harvester 0.6.94 (Earl and vonHoldt 2012). The result was plotted with Distruct 1.1 (Rosenberg 2004). As the program Structure assumes Hardy–Weinberg equilibrium, which may not hold in potentially selfing *Euphrasia* species, we analysed the data also with InStruct (Gao *et al.* 2007). As the best supported grouping was *K* = 1, which in light of results from other methods (see Results) seems overly conservative, and groupings with *K* > 1 failed to yield biologically meaningful results (all but one gene pool were essentially empty, i.e. only minimal proportions of some individuals were assigned to those; data not shown), we did not pursue InStruct analyses any further.

## Results

### Flow cytometry

The FCM analysis yielded two distinct groups of relative DNA amount **[see****Supporting Information—Fig. S1****]**. Taking into account available information on ploidy level in the investigated *Euphrasia* species (Ehrendorfer and Vitek 1984; Vitek 1985), we consider these two size classes to correspond to diploids and tetraploids, respectively. Consequently, 92 individuals were identified as diploids, and 235 individuals were identified as tetraploids. Among the diploids, 28 individuals are *E. inopinata* (12 individuals from the locus classicus and 16 individuals from the neighbouring valley south of the village Vent, both Ötztal) and 59 individuals are *E. sinuata* (17 individuals from the locus classicus in Rofan Mountains, and 42 individuals from Kitzbühler Horn). In addition, five individuals initially determined as *E. minima* turned out to be diploids (hereinafter referred to as *E.* cf. *minima* 2x). Relative genome sizes of the three diploid entities overlapped strongly and showed no interpretable pattern. Tetraploid *E. minima* was found to co-occur with *E.* cf. *minima* 2x, both populations of *E. inopinata*, and the western population of *E. sinuata* (Rofan Mountains), but was absent from the eastern population of *E. sinuata* (Kitzbühler Horn).

### Molecular data

**Figure 2.**
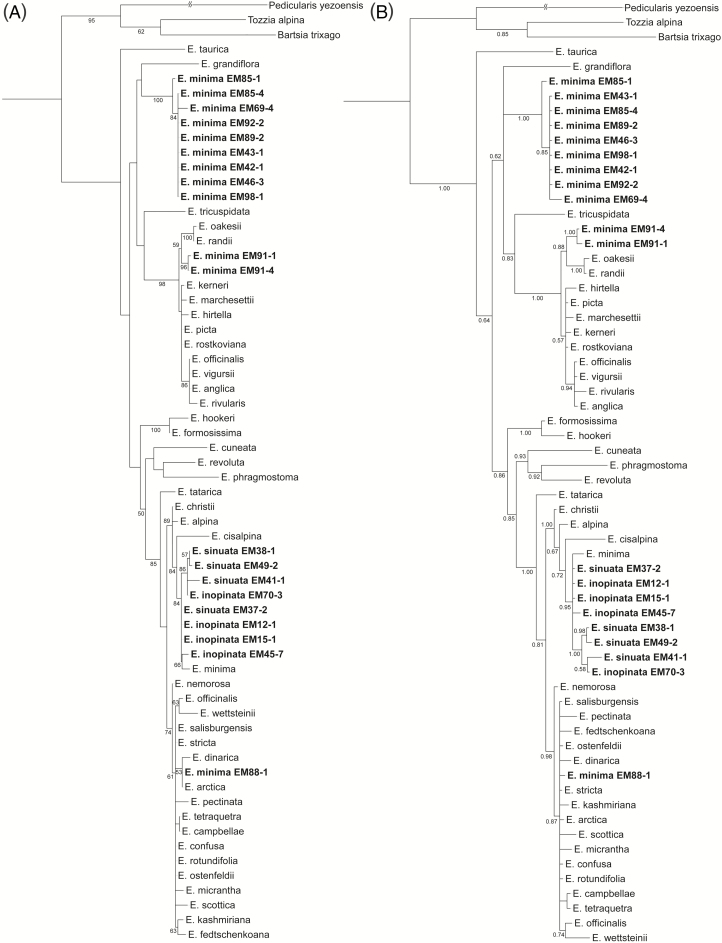
Phylogenetic placement of *Euphrasia minima*, *E. sinuata* and *E. inopinata* based on nuclear ITS sequences analysed using (A) maximum likelihood (numbers at nodes are bootstrap support values of at least 50) and (B) Bayesian inference (majority rule consensus tree, numbers at nodes are posterior probabilities of at least 0.5). Newly obtained sequences are marked in bold.

The newly obtained ITS sequences are available from GenBank **[see****Supporting Information—Table S1****]**. In both ML and BI analyses *E. inopinata* and *E. sinuata* were placed within *Euphrasia* series *Alpinae* (bootstrap support [BS] 89, posterior probability [PP] 1.00; Fig. 2). Samples of *E. minima* did not form a monophyletic group, but were found in four clades. Most individuals were grouped in a distinct clade (BS 100, PP 1.00) of unclear relationship. Two individuals (EM91-1 and EM91-4; BS 96, PP 1.00) were inferred as sister to *E. oakesii* and *E. randii*, yet this group received only limited support (BS 59, PP 0.88); together these were sister (BS 98, PP 1.00) to a hardly supported clade (BS < 50, PP 0.57) comprising species of series *Euphrasia*, such as *E. hirtella* and *E. officinalis*. One individual of *E. minima* (EM88-1) was clustered with a group of Palaearctic species (including, among others, *E. nemorosa* or *E. arctica*; BS 74, PP 0.98). Finally, one individual (the one used in Gussarova *et al.* 2008) grouped with *E. inopinata* and *E. sinuata* (BS 84, PP 0.95).

**Figure 3.**
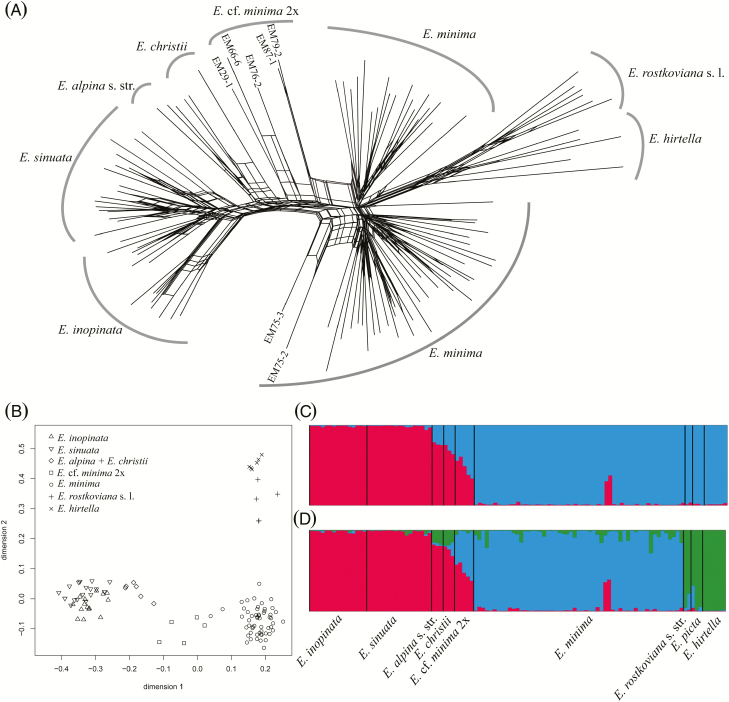
Genetic structure of *Euphrasia inopinata*, *E. sinuata* and related species estimated from AFLP fingerprint data. (A) Neighbour-net based on Jaccard distances (intermediate individuals discussed in the text are indicated); (B) scatter-plot of the first two coordinates of a PCoA using Jaccard distances; (C, D) population structure estimated using the Bayesian clustering approach implemented in Structure at (C) *K* = 2 and (D) *K* = 3.

With the three AFLP primer combinations 343 fragments sized from 141 to 477 bp were successfully scored in 119 individuals, resulting in on average 63 bands per individual. The error rate estimated among replicated individuals was 4.58 %. The neighbour-net revealed three main groups (Fig. 3A). The first group (referred to as Parviflora Group) contained all tetraploid *E. minima*, the second group (referred to as Grandiflora Group) contained the diploids *E. hirtella* and *E. officinalis* s. l. Two individuals of tetraploid *E. minima* (EM75-3 and EM75-2) were clearly separated from the Parviflora Group. The third group (referred to as Alpina Group) contained the diploids *E. alpina*, *E. christii*, *E. inopinata* and *E. sinuata*. Whereas each population of *E. inopinata* and *E. sinuata* was found to be distinct, there was no split supporting the separation of the two species. The individuals of *E.* cf. *minima* 2x (EM76-2, EM66-6, EM29-1, EM87-1 and EM79-2) were situated between the Alpina Group on one side and the Parviflora Group and the Grandiflora Group on the other side. The same three groups were identified by PCoA (Fig. 3B), where the Alpina Group was separated from others along the first axis (25.9 % of the total variance) and the Parviflora Group was separated from the Grandiflora Group along the second axis (9.9 % of the total variance). All individuals inferred to occupy intermediate positions in the neighbour-net (EM75-2, EM75-3, EM76-2, EM66-6, EM29-1, EM87-1 and EM79-2) were positioned between the Alpina Group and the Parviflora Group.

The Delta *K* method suggested *K* = 2 as the optimal number of groups **[see****Supporting Information—Fig. S2B****]**, corresponding to the Alpina Group versus the Grandiflora Group plus Parviflora Group (Fig. 3C). All individuals of *E. minima* inferred to occupy intermediate positions in the neighbour-net (tetraploid EM75-2, EM75-3; diploid EM29-1, EM66-6, EM76-2, EM79-2 and EM87-1) showed strong admixture. Additionally, individuals of *E. alpina* s. l. were inferred to be admixed as well, with the proportion of the minority cluster being between 21 and 36 % (Fig. 3C). If taking the likelihood distribution over different values of *K* into account, *K* = 3 was suggested by a stable likelihood maximum and a relatively high Δ*K* value **[see****Supporting Information—Fig. S2****]**. Under *K* = 3, the Grandiflora Group and the Parviflora Group were separated (Fig. 3D). Patterns of inferred admixture were the same for *K* = 2 and *K* = 3, with all individuals inferred to occupy intermediate positions in the neighbour-net being admixed in roughly equal proportions between the Parviflora and the Alpina Groups and the individuals of *E. alpina* s. l. being admixed between the Alpina and the Grandiflora Groups (Fig. 3D).

## Discussion

On the basis of morphology, Ehrendorfer and Vitek (1984) proposed *E. inopinata* and *E. sinuata* as diploid representatives of *Euphrasia* series *Parviflorae*, including the tetraploid *E. minima*. However, molecular data are inconsistent with their hypothesis. Instead, both ITS sequence and AFLP fingerprint data clearly indicate that *E. inopinata* and *E. sinuata* are not closely related to the morphologically very similar *E. minima*, but instead to *E. alpina* s. l. (Figs 2 and 3). Morphological traits in *Euphrasia* species are highly variable and prone to ecological convergence (Vitek 1998; Twyford *et al.* 2018), explaining the taxonomic misplacement of *E. inopinata* and *E. sinuata* in series *Parviflorae*. In the light of our results, *E. inopinata* and *E. sinuata* need to be taxonomically placed in *Euphrasia* series *Alpinae*.

The phylogenetic placement of *E. inopinata* and *E. sinuata* and their distribution close to the north-eastern edge of the distribution area of *E. alpina* s. l. (Fig. 1; Vitek 1985) suggest that they are peripheral segregates of *E. alpina* s. l. This is in line with the hypothesis of Vitek (1986) that *E. inopinata* and *E. sinuata* are dwarfish derivatives of a more widespread large-flowered diploid, even if this is without elevational differentiation as originally envisioned by Vitek (1986). Although currently lacking from the Austrian Alps (the species is found in the adjacent Italian parts of the Ötztaler Alps: www.florafauna.it, assessed on 10 October 2018), *E. alpina* s. l. or its ancestor has at one point reached the Tyrolean Alps. In peripheral populations, lack of pollinators, especially in the alpine zone, and lack of mates trigger the evolution of self-pollination (Kalisz *et al.* 2004), resulting in the reduction of corolla size (<5 mm in *E. inopinata* and *E. sinuata*: Ehrendorfer and Vitek 1984). Although no pollination data are available for *E. inopinata* and *E. sinuata*, small flower size is a good indicator of increased selfing in *Euphrasia* (French *et al.* 2005). Fixation of thus deviating morphological traits and, in case of *E. sinuata*, also ecological traits with respect to substrate type (siliceous in *E. alpina* s. l. and *E. inopinata* versus limestone and dolomites in *E. sinuata*) is expected to have been fostered by small population sizes, enhancing genetic drift, and by geographic isolation, reducing gene flow from core populations. These shifts and thus the origin of *E. inopinata* and *E. sinuata* might be as recent as the postglacial as suggested for *Euphrasia* species endemic to the British Isles (French *et al.* 2008). As only one population of *E. alpina* s. l. was included, further sampling will be necessary to fully address the evolutionary path from the widespread allogamous *E. alpina* s. l. to the narrowly distributed autogamous *E. inopinata* and *E. sinuata*.

*Euphrasia minima* was hypothesized to be of allotetraploid origin (Yeo 1978; Ehrendorfer and Vitek 1984; Vitek 1986). One of the suggested parental species is *E. hirtella* (Vitek 1986). This is in agreement with our AFLP data (Fig. 3), which indicates a closer relationship between series *Parviflorae* and *Euphrasia*, the latter including *E. hirtella*. As second suggested parental species either *E. inopinata* and *E. sinuata* (Ehrendorfer and Vitek 1984) or *E. alpina* s. l., all from series *Alpinae* in its emended circumscription, were suggested, but neither finds support from the molecular data. After re-examination of the voucher specimen (from Goldberggruppe in the Hohe Tauern range, ~67 km south-east of Kitzbühler Horn), the single accession of *E. minima* used by Gussarova *et al.* (2008) grouping with *E. alpina* in the ITS tree (Fig. 2) might actually be *E. sinuata*. Thus, *E. sinuata* may be more widespread on base-rich soils in the central Alps of Austria, but has remained overlooked due to confusion with the morphologically extremely plastic *E. minima*. The distinctness of a clade of *E. minima* in the ITS tree (Fig. 2) suggests that the origin of this species might be old relative to the species divergence in European *Euphrasia* (including the origin of *E. inopinata* and *E. sinuata*), although peculiarities of ITS evolution (Álvarez and Wendel 2003) warrant caution in inference of (absolute or relative) temporal evolution. Another complicating factor is the high frequency of hybridization in *Euphrasia* (Yeo 1978), outcrossing species of series *Alpinae* (*E. alpina* s. l.) and ser. *Euphrasia* (*E. officinalis* s. l., *E. hirtella*) being no exception (Yeo 1976; Liebst and Schneller 2005). Hybridization and introgression in conjunction with concerted evolution likely explain the phylogenetic position of EM88-1, EM91-1 and EM91-4 off all other accessions of *E. minima* as inferred from ITS data and the two admixed individuals of *E. minima* (EM75-2, EM75-3) inferred from AFLP data. The identity of diploid individuals referred to as *E.* cf. *minima* 2x (EM29-1, EM66-6, EM76-2, EM87-1 and EM79-2) remains unclear, as introgression from *E. minima*, as suggested by AFLP data, appears unlikely given the implied directionality of gene flow from a tetraploid into a diploid (but see Yeo 1956). Instead, these individuals may be hybrids involving any of the widespread and allogamous diploid species *E. alpina*, *E. hirtella* and *E. officinalis* s. l., whose intraspecific genetic diversities certainly are insufficiently covered in this study.

## Conclusion

Molecular data provide clear evidence that diploid *E. inopinata* and *E. sinuata*, narrowly distributed in the central eastern Alps, are peripheral autogamous segregates of the widespread allogamous *E. alpina* s. str. instead of close relatives or ancestors of the morphologically very variable allotetraploid autogamous *E. minima*. This indicates that the shift to autogamy in *E. inopinata* and *E. sinuata* on the one hand and in *E. minima* on the other hand has happened independently, rendering this a well-suited system to study shifts in pollination (allo- to autogamy) in the context of different ploidy levels. The origin of species in formerly glaciated areas, if not associated with major range shifts (colonization from periglacial refugia and subsequent extirpation in those refugia, as has been suggested for members of the *Galium pusillum* complex/Rubiaceae: Kolář *et al.* 2015), commonly involves allopolyploidy and/or hybridization (e.g. *Saxifraga opdalensis*/Saxifragaceae and *Arabidopsis suecica*/Brassicaceae: Steen *et al.* 2000; Jakobsson *et al.* 2006). The herein investigated species *E. inopinata* and *E. sinuata* deviate from this pattern, as both are diploid and show no signs of hybridization with other taxa, rendering them, together with a few other groups, such as *Odontites vernus* and *O. litoralis* (Orobanchaceae), excellent model systems for (possibly postglacial) speciation at the diploid level in formerly heavily glaciated areas.

## Data

Internal transcribed spacer (ITS) sequences are available from GenBank under accession numbers MK040308–MK040324 and MK040326–MK040328. Sequence alignments (as nexus file) and amplified fragment length polymorphism (AFLP) data (as fasta file) are available from Dryad under doi:10.5061/dryad.2pm2j8j.

## Supporting Information

The following additional information is available in the online version of this article—

**Figure S1.** Relative DNA amount of *Euphrasia minima*, *E. inopinata*, *E. sinuata* and *E.* cf. *minima* 2x.

**Figure S2.** Plots of (A) mean Log_e_(X|*K*) and standard deviation over 10 runs and (B) Delta *K* for different *K* values.

**Table S1.** Details of samples: sampling regions, ploidy level, GenBank accession numbers and voucher information.

## Sources of Funding

Financial support from the program of China Scholarships Council (no. 201306860003) to D.P. is acknowledged. Open access funding provided by University of Vienna.

## Contributions by the Authors

Conceptualization: D.P., G.M.S.; formal analysis: D.P.; investigation: D.P., P.S., T.M.; supervision: G.M.S.; writing: D.P., P.S., T.M., E.V., G.M.S.

## Conflict of Interest

None declared.

## Supplementary Material

Supplementary Table S1

Supplementary Material
