# Supplementary material for: Ancestral remnants or peripheral segregates? Phylogenetic relationships of two narrowly endemic Euphrasia species (Orobanchaceae) from the eastern European Alps
Source: AoB Plants. 2019 Feb 19;11(2):plz007. doi: 10.1093/aobpla/plz007 (PMC6435497; doi:10.1093/aobpla/plz007)
Supplement: Supplementary Table S1 [file plz007_suppl_supplementary_table_s1.docx]

Table 1 Sampling locations of investigated *Euphrasia* species (see Supporting Information Table S1 for detailed information).

| Species | Location No. | Region^1^ | Latitude/longitude | Herbarium |
| --- | --- | --- | --- | --- |
| *E. minima* | 1 | I, Alpi Graie: Vallone di Laures | 45°41'13″/7°24'25″ | WU:GMS-266 |
|  | 2 | CH, Alpi Lepontine: Gruppo del Monte Leone | 46°15'27″/8°03'57″ | WU:GMS-267 |
|  | 3 | CH, Alpi Lepontine: Alpi Ticinesi | 46°26'41″/8°30'15″ | WU:GMS-269 |
|  | 4 | CH, Glarner Alpen | 46°58'18″/9°23'50″ | WU:GMS-268 |
|  | 5 | I, Alpi Venoste/Ötztaler Alpen | 46°49'12″/10°41'53″ | WU:GMS-270 |
|  | 6 | I, Alpi Venoste/Ötztaler Alpen | 46°45'20″/10°49'09″ | WU:GMS-264 |
|  | 7 | A, Ötztaler Alpen | 46°49'07″/10°54'02″ | WU:GMS-256 |
|  | 8 | A, Ötztaler Alpen | 47°51'57″/11°01'24″ | NHM2014-0014158 |
|  | 9 | I, Alpi Venoste/Ötztaler Alpen | 46°51'23″/11°05'37″ | WU:GMS-261 |
|  | 10 | A, Stubaier Alpen | 47°06'57″/11°11'49″ | WU:GMS-273 |
|  | 11 | A, Rofangebirge und Brandenberger Alpen | 47°26'37″/11°45'54″ | WU:GMS-274 |
|  | 12 | A, Hohe Tauern: Venedigergruppe & Lasörlinggruppe | 47°00'04″/12°15'10″ | WU:GMS-272 |
| *E. sinuata* | 11 | A, Rofangebirge und Brandenberger Alpen | 47°26'37″/11°45'54″ | WU:GMS-251 |
|  | 13 | A, Kitzbüheler Alpen | 47°28'22″/12°25'49″ | NHM2014-0014161 |
| *E. inopinata* | 7 | A, Ötztaler Alpen | 46°49'07″/10°54'02″ | WU:GMS-249 |
|  | 8 | A, Ötztaler Alpen | 47°51'57″/11°01'24″ | NHM2014-0014157 |
| *E.* cf. *minima* 2x | 2 | CH, Alpi Lepontine: Gruppo del Monte Leone | 46°15'27″/8°03'57″ | WU:GMS-255 |
|  | 6 | I, Alpi Venoste/Ötztaler Alpen | 46°45'20″/10°49'09″ | WU:GMS-254 |
|  | 7 | A, Ötztaler Alpen | 46°49'07″/10°54'02″ | WU:GMS-253 |
| *E. alpina* s. str. | 1 | I, Alpi Graie: Vallone di Laures | 45°41'13″/7°24'25″ | WU:GMS-276 |
| *E. christii* | 1 | I, Alpi Graie: Vallone di Laures | 45°41'13″/7°24'25″ | WU:GMS-277 |
| *E. rostkoviana* s. l. | 8 | A, Ötztaler Alpen | 46°51'57″/11°01'24″ | NHM2014-0014158 |
| *E. hirtella* | 11 | A, Rofangebirge und Brandenberger Alpen | 47°26'37″/11°45'54″ | NHM2014-0014155 |

^1^ I = Italy; CH = Switzerland; A = Austria
