# Supplementary material for: Ancestral remnants or peripheral segregates? Phylogenetic relationships of two narrowly endemic Euphrasia species (Orobanchaceae) from the eastern European Alps
Source: AoB Plants. 2019 Feb 19;11(2):plz007. doi: 10.1093/aobpla/plz007 (PMC6435497; doi:10.1093/aobpla/plz007)
Supplement: Supplementary Material [file plz007_suppl_supplementary_material.pdf]

Figure S1 Relative DNA amount of *E. minima*, *E. inopinata*, *E. sinuata* and *E.* cf. *minima* 2x individuals. In each panel one taxon is highlighted in black.

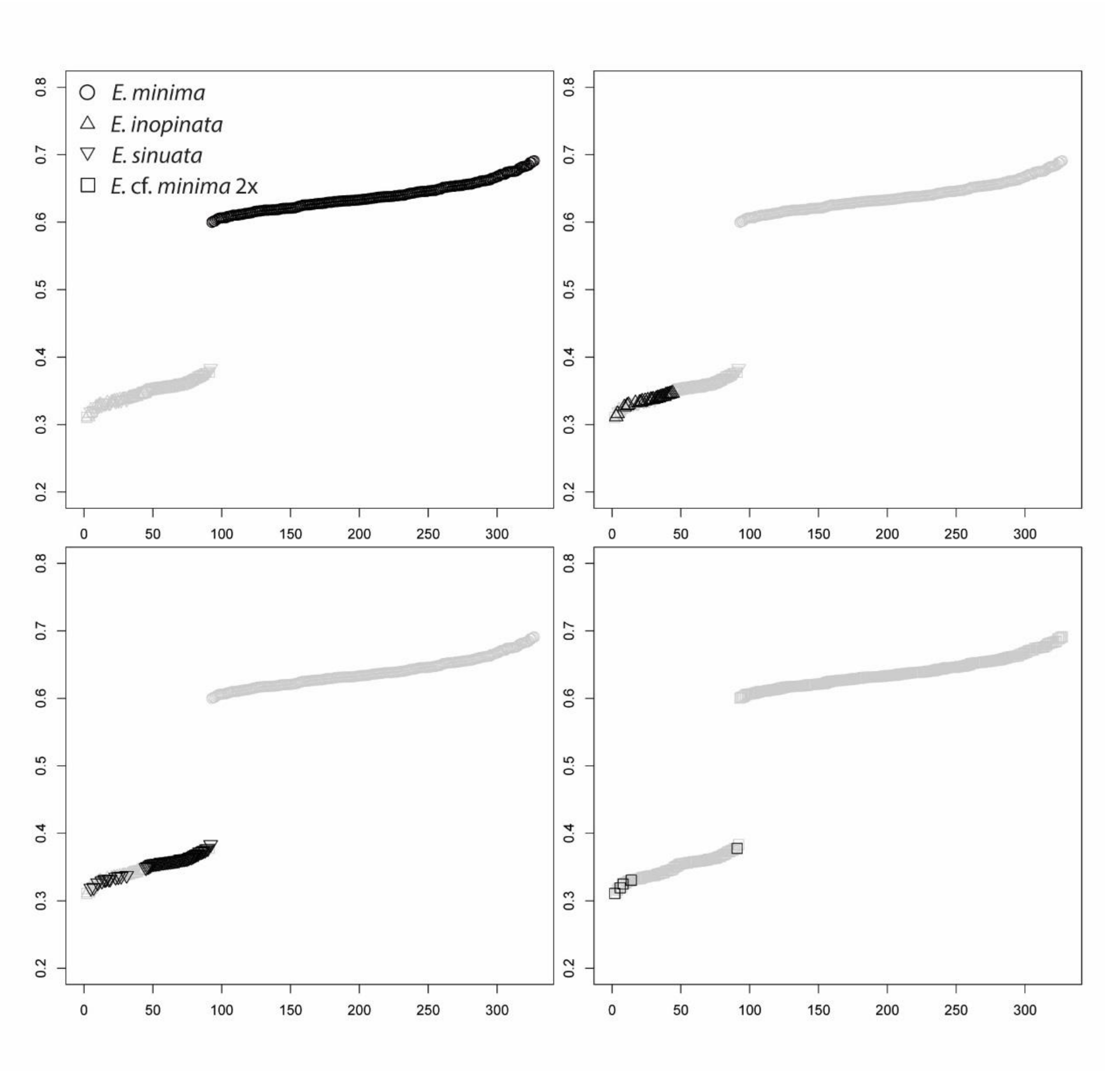

Figure S2 Plots of (a) mean $\text{Log}_e(X|K)$ and standard deviation over 10 runs and (b) Delta K for each K value.

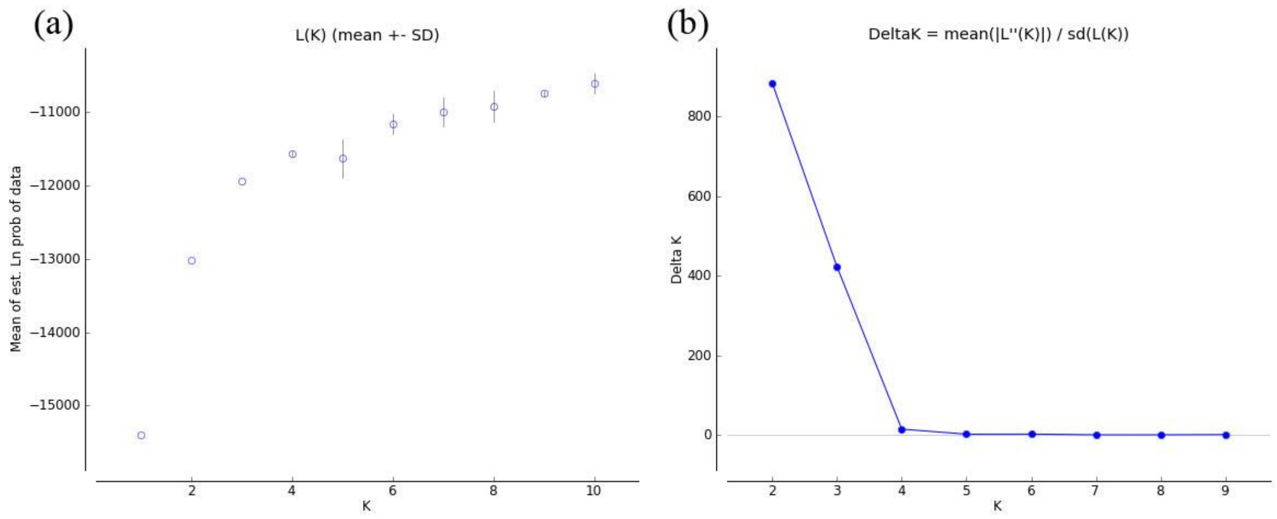

Table S1 Detail of samples: sampling regions, ploidy level, GenBank accession numbers and voucher information.

Species	Location No.	Sampling region[1]	Latitude/longitude	Herbarium	Population	Individual	Ploidy level	GenBank accession number
E. inopinata	8	A, Ötztaler Alpen	47°51'57″/11°01'24″	NHM2014-0014157	EM45	EM45-1	2*x*	
						EM45-2	2*x*	
						EM45-3	2*x*	
						EM45-4	2*x*	
						EM45-5	2*x*	
						EM45-6	2*x*	
						EM45-7	2*x*	MK040313
						EM45-8	2*x*	
						EM45-9	2*x*	
						EM45-10	2*x*	
						EM45-11	2*x*	
						EM45-12	2*x*	
	7	A, Ötztaler Alpen	46°49'07″/10°54'02″	WU:GMS-249	EM12	EM12-1	2*x*	MK040308
						EM12-2	2*x*	
			46°49'01″/10°53'58″	WU:GMS-249	EM14	EM14-1	2*x*	
			46°49'07″/10°53'58″	WU:GMS-249	EM15	EM15-1	2*x*	MK040309
						EM15-2	2*x*	
			46°47'21″/10°51'69″	WU:GMS-250	EM66	EM66-4	2*x*	
			46°48'18″/10°53'26″	WU:GMS-250	EM69	EM69-1	2*x*	
						EM69-2	2*x*	
						EM69-3	2*x*	
			46°48'49″/10°53'49″	WU:GMS-250	EM70	EM70-1	2*x*	
						EM70-2	2*x*	
						EM70-3	2*x*	MK040315
						EM70-4	2*x*	

						EM70-5	2*x*	
						EM70-6	2*x*	
			46°48'57″/10°53'52″	WU:GMS-250	EM71	EM71-1	2*x*	
E. sinuata	13	A, Kitzbüheler Alpen	47°28'22″/12°25'49″	NHM2014-0014161	EM37	EM37-1	2*x*	
						EM37-2	2*x*	MK040310
						EM37-3	2*x*	
						EM37-4	2*x*	
						EM37-5	2*x*	
						EM37-6	2*x*	
						EM37-7	2*x*	
						EM37-8	2*x*	
			47°28'26″/12°25'41″	NHM2014-0014161	EM38	EM38-1	2*x*	MK040311
						EM38-2	2*x*	
						EM38-3	2*x*	
						EM38-4	2*x*	
						EM38-5	2*x*	
						EM38-6	2*x*	
						EM38-7	2*x*	
						EM38-8	2*x*	
						EM38-9	2*x*	
						EM38-10	2*x*	
						EM38-11	2*x*	
						EM38-12	2*x*	
						EM38-13	2*x*	
						EM38-14	2*x*	
						EM38-15	2*x*	
						EM38-16	2*x*	
						EM38-17	2*x*	
						EM38-18	2*x*	

		47°28'26″/12°25'41″	NHM2014-0014161	EM41	EM41-1	2*x*	MK040312
					EM41-2	2*x*	
					EM41-3	2*x*	
					EM41-4	2*x*	
					EM41-5	2*x*	
					EM41-6	2*x*	
					EM41-7	2*x*	
					EM41-8	2*x*	
					EM41-9	2*x*	
					EM41-10	2*x*	
					EM41-11	2*x*	
					EM41-12	2*x*	
					EM41-13	2*x*	
					EM41-14	2*x*	
					EM41-15	2*x*	
		47°28'22″/12°25'44″	NHM2014-0014161	EM61	EM61-4	2*x*	
11	A, Rofangebirge und Brandenberger Alpen	47°26'37″/11°45'54″	WU:GMS-251	EM46	EM46-1	2*x*	
					EM46-4	2*x*	
		47°26'37″/11°45'54″	WU:GMS-251	EM49	EM49-1	2*x*	
					EM49-2	2*x*	MK040314
					EM49-3	2*x*	
					EM49-4	2*x*	
					EM49-5	2*x*	
					EM49-6	2*x*	
					EM49-7	2*x*	
					EM49-8	2*x*	
		47°27'16″/11°47'08″	WU:GMS-252	EM96	EM96-1	2*x*	
					EM96-2	2*x*	

						EM96-3	2*x*
			47°27'04″/11°46'47″	WU:GMS-252	EM97	EM97-2	2*x*
			47°27'49″/11°46'30″	WU:GMS-252	EM98	EM98-2	2*x*
						EM98-4	2*x*
			47°26'43″/11°46'00″	WU:GMS-252	EM99	EM99-6	2*x*
E. cf. *minima*	7	A, Ötztaler Alpen	46°47'21″/10°51'69″	WU:GMS-253	EM66	EM66-6	2*x*
	6	I, Alpi Venoste/Ötztaler Alpen	46°45'20″/10°49'09″	WU:GMS-254	EM29	EM29-1	2*x*
			46°45'43″/10°48'56″	WU:GMS-278	EM76	EM76-2	2*x*
			46°45'08″/10°49'07″	WU:GMS-278	EM79	EM79-2	2*x*
	2	CH, Alpi Lepontine: Gruppo del Monte Leone	46°15'27″/8°03'57″	WU:GMS-255	EM87	EM87-1	2*x*
E. minima	7	A, Ötztaler Alpen	46°51'19″/10°54'24″	WU:GMS-256	EM1	EM1-1	4*x*
						EM1-2	4*x*
						EM1-3	4*x*
			46°51'21″/10°53'58″	WU:GMS-256	EM2	EM2-1	4*x*
						EM2-3	4*x*
			46°50'41″/10°51'57″	WU:GMS-256	EM3	EM3-1	4*x*
						EM3-2	4*x*
			46°50'41″/10°51'57″	WU:GMS-256	EM4	EM4-1	4*x*
						EM4-2	4*x*
			46°50'34″/10°51'47″	WU:GMS-256	EM5	EM5-1	4*x*
						EM5-2	4*x*
						EM5-3	4*x*
						EM5-4	4*x*
			46°50'06″/10°51'03″	WU:GMS-256	EM6	EM6-1	4*x*
						EM6-2	4*x*
			46°51'18″/10°54'36″	WU:GMS-256	EM7	EM7-1	4*x*
						EM7-2	4*x*
			46°52'05″/10°53'06″	WU:GMS-257	EM8	EM8-1	4*x*
						EM8-2	4*x*

	46°52'05″/10°52'40″	WU:GMS-257	EM9	EM9-1	4*x*	
				EM9-2	4*x*	
	46°52'02″/10°53'01″	WU:GMS-257	EM10	EM10-1	4*x*	
	46°51'20″/10°54'39″	WU:GMS-258	EM11	EM11-1	4*x*	
	46°49'07″/10°54'02″	WU:GMS-258	EM13	EM13-1	4*x*	
				EM13-2	4*x*	
				EM13-3	4*x*	
				EM13-4	4*x*	
	46°50'15″/10°54'48″	WU:GMS-258	EM16	EM16-1	4*x*	
	46°47'21″/10°51'69″	WU:GMS-259	EM66	EM66-1	4*x*	
				EM66-2	4*x*	
				EM66-3	4*x*	
				EM66-5	4*x*	
	46°47'37″/10°52'09″	WU:GMS-259	EM67	EM67-1	4*x*	
				EM67-2	4*x*	
				EM67-3	4*x*	
				EM67-4	4*x*	
	46°47'59″/10°52'53″	WU:GMS-259	EM68	EM68-1	4*x*	
				EM68-2	4*x*	
				EM68-3	4*x*	
				EM68-4	4*x*	
				EM68-5	4*x*	
				EM68-6	4*x*	
	46°48'18″/10°53'26″	WU:GMS-259	EM69	EM69-4	4*x*	MK040319
	46°49'15″/10°54'02″	WU:GMS-259	EM72	EM72-1	4*x*	
				EM72-2	4*x*	
				EM72-3	4*x*	
				EM72-4	4*x*	
				EM72-5	4*x*	
				EM72-6	4*x*	
	46°49'57″/10°54'34″	WU:GMS-259	EM73	EM73-1	4*x*	

					EM73-2	4*x*	
					EM73-3	4*x*	
					EM73-4	4*x*	
8	A, Ötztaler Alpen	46°51'57″/11°01'24″	NHM2014-0014158	EM42	EM42-1	4*x*	MK040316
					EM42-2	4*x*	
					EM42-3	4*x*	
					EM42-4	4*x*	
					EM42-5	4*x*	
					EM42-6	4*x*	
					EM42-7	4*x*	
		46°51'57″/11°01'24″	NHM2014-0014158	EM43	EM43-1	4*x*	MK040317
					EM43-2	4*x*	
					EM43-3	4*x*	
					EM43-4	4*x*	
					EM43-5	4*x*	
					EM43-6	4*x*	
					EM43-7	4*x*	
					EM43-8	4*x*	
					EM43-9	4*x*	
9	I, Alpi Venoste/Ötztaler Alpen	46°51'23″/11°05'37″	WU:GMS-261	EM17	EM17-1	4*x*	
					EM17-2	4*x*	
		46°51'33″/11°05'41″	WU:GMS-261	EM18	EM18-1	4*x*	
					EM18-2	4*x*	
		46°52'27″/11°06'17″	WU:GMS-261	EM19	EM19-1	4*x*	
					EM19-2	4*x*	
		46°52'43″/11°06'32″	WU:GMS-261	EM20	EM20-1	4*x*	
					EM20-2	4*x*	
		46°53'56″/11°10'50″	WU:GMS-262	EM21	EM21-1	4*x*	

					EM21-2	4*x*
					EM21-3	4*x*
					EM21-4	4*x*
					EM21-5	4*x*
		46°54'06″/11°11'11″	WU:GMS-262	EM23	EM23-1	4*x*
					EM23-2	4*x*
		46°54'34″/11°11'34″	WU:GMS-262	EM24	EM24-2	4*x*
		46°54'47″/11°11'26″	WU:GMS-262	EM25	EM25-1	4*x*
					EM25-2	4*x*
		46°53'08″/11°10'20″	WU:GMS-262	EM26	EM26-1	4*x*
					EM26-2	4*x*
		46°47'24″/11°06'03″	WU:GMS-263	EM27	EM27-1	4*x*
					EM27-2	4*x*
					EM27-3	4*x*
					EM27-5	4*x*
6	A, Ötztaler Alpen	46°45'07″/10°49'11″	WU:GMS-264	EM28	EM28-1	4*x*
					EM28-2	4*x*
					EM28-3	4*x*
					EM28-4	4*x*
					EM28-6	4*x*
					EM28-7	4*x*
		46°45'34″/10°49'07″	WU:GMS-264	EM30	EM30-1	4*x*
		46°45'42″/10°48'55″	WU:GMS-264	EM31	EM31-1	4*x*
		46°46'09″/10°48'33″	WU:GMS-265	EM74	EM74-1	4*x*
					EM74-2	4*x*
					EM74-3	4*x*
					EM74-4	4*x*
					EM74-5	4*x*
		46°45'56″/10°48'40″	WU:GMS-265	EM75	EM75-1	4*x*
					EM75-2	4*x*
					EM75-3	4*x*

		46°45'43″/10°48'56″	WU:GMS-265	EM76	EM76-1	4*x*	
					EM76-3	4*x*	
					EM76-4	4*x*	
					EM76-5	4*x*	
					EM76-6	4*x*	
		46°45'32″/10°49'02″	WU:GMS-265	EM77	EM77-1	4*x*	
					EM77-2	4*x*	
					EM77-4	4*x*	
		46°45'22″/10°49'04″	WU:GMS-265	EM78	EM78-1	4*x*	
					EM78-2	4*x*	
		46°45'08″/10°49'07″	WU:GMS-265	EM79	EM79-3	4*x*	
					EM79-4	4*x*	
1	I, Alpi Graie: Vallone di Laures	45°41'25″/7°24'16″	WU:GMS-266	EM91	EM91-1	4*x*	MK040327
					EM91-3	4*x*	
					EM91-4	4*x*	MK040328
		45°41'38″/7°24'32″	WU:GMS-266	EM92	EM92-1	4*x*	
					EM92-2	4*x*	MK040323
					EM92-3	4*x*	
					EM92-4	4*x*	
		45°41'44″/7°24'26″	WU:GMS-266	EM93	EM93-1	4*x*	
					EM93-2	4*x*	
					EM93-3	4*x*	
					EM93-4	4*x*	
2	CH, Alpi Lepontine: Gruppo del Monte Leone	46°15'27″/8°03'57″	WU:GMS-267	EM87	EM87-2	4*x*	
					EM87-3	4*x*	
		46°15'14″/8°03'58″	WU:GMS-267	EM88	EM88-1	4*x*	MK040322
					EM88-2	4*x*	
					EM88-3	4*x*	
					EM88-4	4*x*	

		46°15'01″/8°03'47″	WU:GMS-267	EM89	EM89-1	4*x*	
					EM89-2	4*x*	MK040326
					EM89-3	4*x*	
					EM89-4	4*x*	
		46°14'55″/8°03'31″	WU:GMS-267	EM90	EM90-1	4*x*	
					EM90-2	4*x*	
					EM90-3	4*x*	
4	CH, Glarner Alpen	46°58'18″/9°23'50″	WU:GMS-268	EM94	EM94-1	4*x*	
					EM94-2	4*x*	
					EM94-3	4*x*	
					EM94-4	4*x*	
		46°58'24″/9°24'16″	WU:GMS-268	EM95	EM95-1	4*x*	
					EM95-2	4*x*	
					EM95-3	4*x*	
					EM95-4	4*x*	
3	CH, Alpi Lepontine: Alpi Ticinesi	46°26'41″/8°30'15″	WU:GMS-269	EM85	EM85-1	4*x*	MK040324
					EM85-2	4*x*	
					EM85-3	4*x*	
					EM85-4	4*x*	MK040321
		46°26'25″/8°29'01″	WU:GMS-269	EM86	EM86-1	4*x*	
					EM86-2	4*x*	
					EM86-3	4*x*	
					EM86-4	4*x*	
					EM86-5	4*x*	
5	I, Alpi Venoste/Ötztaler Alpen	46°49'12″/10°41'53″	WU:GMS-270	EM32	EM32-1	4*x*	
		46°49'10″/10°41'58″	WU:GMS-270	EM33	EM33-1	4*x*	
					EM33-2	4*x*	
		46°49'07″/10°42'31″	WU:GMS-270	EM34	EM34-1	4*x*	
					EM34-2	4*x*	

					EM34-3	4*x*
					EM34-4	4*x*
					EM34-5	4*x*
					EM34-6	4*x*
		46°49'12″/10°42'43″	WU:GMS-270	EM35	EM35-1	4*x*
					EM35-2	4*x*
		46°49'09″/10°42'23″	WU:GMS-270	EM36	EM36-1	4*x*
					EM36-2	4*x*
		46°49'08″/10°42'39″	WU:GMS-271	EM80	EM80-1	4*x*
					EM80-2	4*x*
					EM80-3	4*x*
					EM80-4	4*x*
					EM80-5	4*x*
					EM80-6	4*x*
		46°49'07″/10°42'16″	WU:GMS-271	EM81	EM81-1	4*x*
					EM81-2	4*x*
					EM81-3	4*x*
					EM81-4	4*x*
					EM81-5	4*x*
		46°49'11″/10°41'49″	WU:GMS-271	EM82	EM82-1	4*x*
					EM82-2	4*x*
					EM82-3	4*x*
					EM82-4	4*x*
					EM82-5	4*x*
					EM82-6	4*x*
					EM82-7	4*x*
					EM82-8	4*x*
		46°49'06″/10°41'22″	WU:GMS-271	EM83	EM83-1	4*x*
					EM83-2	4*x*
					EM83-3	4*x*
		46°49'39″/10°40'13″	WU:GMS-271	EM84	EM84-1	4*x*

					EM84-2	4*x*	
					EM84-3	4*x*	
					EM84-4	4*x*	
12	A, Hohe Tauern: Venedigergruppe & Lasörlinggruppe	47°00'04″/12°15'10″	WU:GMS-272	EM59	EM59-1	4*x*	
					EM59-2	4*x*	
10	A, Stubaier Alpen	47°06'57″/11°11'49″	WU:GMS-273	EM63	EM63-1	4*x*	
					EM63-2	4*x*	
					EM63-3	4*x*	
					EM63-4	4*x*	
		47°07'13″/11°12'21″	WU:GMS-273	EM64	EM64-1	4*x*	
					EM64-2	4*x*	
					EM64-3	4*x*	
					EM64-4	4*x*	
		47°07'01″/11°12'33″	WU:GMS-273	EM65	EM65-1	4*x*	
					EM65-2	4*x*	
					EM65-3	4*x*	
					EM65-4	4*x*	
11	A, Rofangebirge und Brandenberger Alpen	47°26'37″/11°45'54″	WU:GMS-274	EM46	EM46-2	4*x*	
					EM46-3	4*x*	MK040318
					EM46-5	4*x*	
					EM46-6	4*x*	
					EM46-7	4*x*	
					EM46-8	4*x*	
					EM46-9	4*x*	
					EM46-10	4*x*	
					EM46-11	4*x*	
					EM46-12	4*x*	
					EM46-13	4*x*	

			47°27′16″/11°47′08″	WU:GMS-275	EM96	EM96-4	4*x*	
			47°27′04″/11°46′47″	WU:GMS-275	EM97	EM97-4	4*x*	
			47°27′49″/11°46′30″	WU:GMS-275	EM98	EM98-1	4*x*	MK040320
						EM98-3	4*x*	
			47°26′43″/11°46′00″	WU:GMS-275	EM99	EM99-1	4*x*	
						EM99-2	4*x*	
						EM99-3	4*x*	
						EM99-4	4*x*	
						EM99-5	4*x*	
E. christii	1	I, Alpi Graie: Vallone di Laures	45°41'13″/7°24'25″	WU:GMS-276	EC	EC-1	-	
						EC-2	-	
						EC-3	-	
E. alpina s. str.	1	I, Alpi Graie: Vallone di Laures	45°41'13″/7°24'25″	WU:GMS-277	EA	EA-1	-	
						EA-2	-	
						EA-3	-	
E. picta	8	A, Ötztaler Alpen	46°51'57″/11°01'24″	NHM2014-0014160	EP	EP-1	-	
						EP-2	-	
						EP-3	-	
E. rostkoviana s. str.	8	A, Ötztaler Alpen	46°51'57″/11°01'24″	NHM2014-0014158	ER	ER-1	-	
						ER-2	-	
E. hirtella	11	A, Rofangebirge und Brandenberger Alpen	47°26'37″/11°45'54″	NHM2014-0014155	EH	EH-1	-	
						EH-2	-	
						EH-3	-	
						EH-4	-	
						EH-5	-	

[1] I = Italy; CH = Switzerland; A = Austria
